# An Integrative Dual-Layer Poly-L-Lactic Acid Fibrous Membrane Prevents Peritendinous Adhesions

**DOI:** 10.3389/fbioe.2020.00387

**Published:** 2020-05-05

**Authors:** Wei Wang, Ning He, Zhixiao Yao, Xu Wang, Hui Wang, Miao He, Yusheng Li, Yun Qian

**Affiliations:** ^1^Department of Orthopedics, Shanghai Sixth People’s Hospital East Affiliated to Shanghai University of Medicine & Health Sciences, Shanghai, China; ^2^Department of Orthopedics, Shanghai Jiao Tong University Affiliated Sixth People’s Hospital, Shanghai, China; ^3^Department of Orthopedics, Shanghai Eighth People’s Hospital, Shanghai, China; ^4^Department of Orthopedics, Xiangya Hospital, Central South University, Changsha, China; ^5^National Clinical Research Center for Geriatric Disorders, Xiangya Hospital, Central South University, Changsha, China

**Keywords:** adhesion, tendon regeneration, tendon gliding, anti-adhesion membrane, tissue engineering

## Abstract

Anti-adhesion membranes are prospective scaffolds for preventing peritendinous adhesion after injury. However, currently available scaffolds have some limitations, such as low efficacy for anti-adhesion, low quality of tendon healing, and unknown drug interactions. Thus, in this study, we designed an innovative structure involving an integrated dual-layer poly(L-lactic acid) (PLLA) electrospun membrane for preventing peritendonous adhesion by promoting tendon gliding. We investigated the surface morphology and wettability of the fiber scaffold. The adhesion and proliferation of fibroblasts were low on the PLLA fibrous membrane. Compared with single-layer membranes, the dual-layer PLLA fiber scaffold reduced adhesion to the tissues. The gliding space persisted until recovery in chicken extensor flexor tendons *in vivo*. Thus, this innovative PLLA membrane scaffold could prevent adhesion and promote gliding to facilitate tendon healing.

## Introduction

Peritendinous adhesion of tendons after hand surgery (Titan et al., 2019) can cause dysfunction of the extremities and requires complicated re-operative surgery. Despite the availability of modern surgical techniques and advanced rehabilitation protocols, peritendinous adhesion remains a challenge for hand surgeons.

Two types of tendon healing, i.e., intrinsic and extrinsic, co-exist after tendon injury. The characteristics of intrinsic healing include tenocyte proliferation and migration into the injury site. In contrast, extrinsic healing occurs via invasion of cells from the surrounding sheath and synovium (Manske and Lesker, 1984). One key approach to preventing malfunction of the extremities caused by peritendinous adhesion is the promotion of tendon gliding after surgery. However, current innovations cannot completely restrict adhesion during extrinsic healing. Furthermore, biochemical drugs applied on membranes (such as ibuprofen, microRNAs, basic fibroblast growth factor, and silver nanoparticles) cause side effects or reduce the quality of tendon healing (Shalumon et al., 2018; Zhou et al., 2018; Chen et al., 2019; Zhao et al., 2019).

Polymer fibrous membranes are promising tools for preventing tendon adhesion (Inui et al., 2010; Li et al., 2017; Liu W. et al., 2020), and electrospinning approaches have been developed for use in the field of polymer application (Farokhi et al., 2020). The beneficial characteristics of poly(L-lactic acid) (PLLA), including viscosity, biodegradability, biocompatibility, and biosafety, have facilitate the application of PLLA in the biomedical field (Kumar et al., 2015; Ge et al., 2018). The PLLA membrane can be an ideal barrier for effective tissue separation with satisfactory biocompatibility (Li et al., 2017; Sensini et al., 2017; Martelli et al., 2020). Moreover, the porous design of this scaffold confers the product with high porosity, large surface area to volume ratio, and small pore size, which helps improve intrinsic regeneration by permitting free exchange of nutrients into the injured area (Landau et al., 2017; Morelli et al., 2019).

In this study, we evaluated the ideal polymer structure for promoting the gliding of repaired tendons. To this end, we designed an integrative dual-layer PLLA membrane using a “gradient dense” method that involved electrospinning. In this method, the two layers of the fibrous membrane were produced successively using ethylalcohol, and integration was achieved by a shearing force, which combined the margins of the two layers into a single layer. We hypothesized that the space left between the two layers could act as an artificial gliding layer during the tendon healing process. The aim of this study was to evaluate whether this novel integrative dual-layer PLLA membrane could prevent tendon adhesion and promote tendon gliding.

## Materials and Methods

### Electrospun Fabrication of Fiber Nanoscaffolds

Poly(L-lactic acid) [molecular weight (Mw) = 50 kDa, Mw/Mn = 1.61] was prepared via bulk ring-opening polymerization of L-lactide using stannous chloride as an initiator (Jinan Daigang Co., Jinan, China). Dichloromethane and trichloromethane were purchased from the Chinese Medicine Group Chemical Reagent Corporation. Dextran (average Mw = 64,000–76,000 Da) and polyethylene glycol (PEG6000) were purchased from Sigma (St. Louis, MO, United States). All other chemicals and solutions were obtained from Guoyao Corporation (Shanghai, China).

Electrospun fabrication was performed as described previously (Cui et al., 2009). A high-voltage (15 kV) direct current was applied to fabricate the PLLA fibrous membrane. The polymeric solution was fed into the needle tip using a syringe pump at an injection rate of 3.0 mL/h. A grounded aluminum foil was used to collect the fibrous membranes. A 15-cm distance was left between the spray head and the collecting device. The first layer was sprayed with ethylalcohol and vacuum-dried for 1 day before collecting the second layer on the same foil. The second layer was sprayed with ethylalcohol and dried under the same conditions as those used for the first layer.

### Surface Morphology and Physical Characteristics of the Fiber Nanoscaffolds

Next, we evaluated the density and porosity of the scaffolds as described previously (Liu et al., 2013). Scanning electron microscopy (SEM; FEI Quanta 200 Scanning Electron Microscope; FEI, Hillsboro, OR, United States) was performed to observe the morphology of the membranes. Water contact angles were evaluated using a contact-angle analyzer (DSA25S; Data Physics Corporation). We captured random images from five replicates for each group at 1000 × magnification. Photoshop 8.0 was used to evaluate the average fiber diameter based on random images from 20 fiber tissues and 200 sections.

### *In vitro* Cell Culture

The adhesive and proliferative states were assessed using chicken embryonic fibroblasts (UMNSAH/DF-1 cells) from the surfaces of the single-layer and dual-layer PLLA fibrous membranes. The cells were incubated in Dulbecco’s modified Eagle’s medium supplemented with 10% fetal bovine serum and antibiotics (100 U/mL penicillin and 100 mg/mL streptomycin) at 37°C in an atmosphere containing 5% CO_2_. The culture medium was replaced three times a week. After reaching confluence, cells were collected by trypsinization with 0.25% trypsin. Residual ethanol was washed out from the scaffolds by immersion in phosphate-buffered saline (PBS) for 90 min. Finally, samples were transferred to a 24-well plate (1 × 10^5^ cells/mL, 100 mL/well).

### Cell Viability Assay

On days 1 and 5 of incubation, 3-(4,5-dimethylthiazol-2-yl)-2,5-diphenyltetrazolium bromide (MTT) solution (5 mg/mL) was loaded in each sample well (100 mL/well), and the samples were incubated at 37°C for 4 h. Subsequently, cells were washed with PBS, and 200 mL of the solution (MTT formazan crystals dissolved in 1 mL dimethyl sulfoxide) from each well was transferred to a 96-well plate. The absorbance of the samples was measured at 490 nm using a microplate reader (Synergy 2; BioTek, Winooski, VT, United States).

### Immunofluorescence Assay

UMNSAH/DF-1 cells in the single-layer and dual-layer scaffolds were detected by fluorescence microscopy. Briefly, cells were cultured for 24 h in different scaffolds at an initial density of 10^5^ cells/mL and then stained with 4′,6-diamidino-2-phenylindole and phalloidin (Sigma) for 30 min. Finally, cell morphology was observed under a fluorescence microscope (Leica, Wetzlar, Germany). Nuclei were stained blue, and the cytoskeleton on the surface of the fibrous membranes was stained red.

### *In vivo* Assays

The animal experiment was approved by the ethical committee of Xiangya Hospital, Central South University (201908798). Leghorn chickens (*n* = 30; weight, 1.5–2.0 kg; provided by the Laboratory Animal Center of Shanghai Institute for Biological Science) were anesthetized by intramuscular administration of ketamine hydrochloride (50 mg/kg). An elastic tourniquet was applied after sterilization of the operation area. The flexor digitorum profundus (FDP) tendon sheath was exposed at the right lateral side of the phalanges from the third toe through a 15-mm incision. The exposed FDP was then dissected in a transverse direction after incising the tendon sheath. The chickens were randomly allocated into the control, single-layer membrane, and dual-layer membrane groups. The PLLA fibrous membranes (single layer and dual layer) were cut into 1 × 1-cm sheets. The damaged tissues were then enclosed with (single-layer and dual-layer groups) or without (control group) fiber scaffolds, the skin was sutured with 6-0 prolene using the modified Kessler technique (Zeng et al., 2003), and the injured leg was immobilized using a splint (Liu et al., 2013).

### Macroscopic Evaluation

The severity of adhesions around the tendons was evaluated using a macro-scale grading system in a semiquantitative manner by two independent researchers [level 1: adhesion free; level 2: slightly separable adhesion; level 3: mildly inseparable adhesion; level 4: moderate adhesion (approximately 35–60% in total tissues); level 5: severe adhesion (>60% in total tissues)] (Cashman et al., 2004).

### Histological Evaluation

The specimens (third toes, including the FDP tendons) were immersed in 4% paraformaldehyde solution overnight, followed by decalcification in 10% EDTA solution at 25°C for 30 days. Samples (4 mm thick) were dehydrated with increasing concentrations of ethanol, embedded in paraffin, and then stained with hematoxylin and eosin and Masson’s trichrome stain. The adhesion condition was defined as follows: level 1, adhesion free; level 2, mild adhesion (<33% in total tissues); level 3, moderate adhesion (33–66% in total tissues); and level 4, severe adhesion (>66% in total tissues). Tendon repair was defined as follows: level 1, excellent (continuous and smooth tendon structure); level 2, good (aligned collagen bundles inside tendons with slight adhesive epitenon); level 3, fair (irregular alignment and discontinuous collagen bundles); level 4, poor (broken and granular deposit) (Liu et al., 2012). Histology slides were prepared using distal, middle, and proximal layers from each sample and observed under a light microscope (Leica DM 4000 B) by independent researchers.

### Biomechanical Assay

The regenerated tendon tissue was exposed at the ankle prior to biomechanical measurements. A rheometer (Instron 5548; Norwood, MA, United States) was used to test the work of flexion and peak tensile strength. The proximal side of the specimen was fixed to a dynamometer, whereas the other side of the sample was placed in a self-made clamp. Load (N) and displacement (mm) were measured to evaluate the work of flexion under 10-mm/min pulling of the FDP tendon until the angle of the proximal interdigital joint reached 40°. The area under the curve of load and displacement represented the work of flexion. Peak tensile strength referred to the tensile strength at which the FDP tendon and sheath separated completely.

### Statistics

The results are expressed as means and standard deviations. Data were analyzed using one-way analysis of variance (SPSS 11.0; IBM Corporation, Armonk, NY, United States). Results with *P* values of less than or equal to 0.05 were regarded as significant.

## Results

### Structural and Physical Properties of the PLLA Fiber Scaffold

**FIGURE 1:**
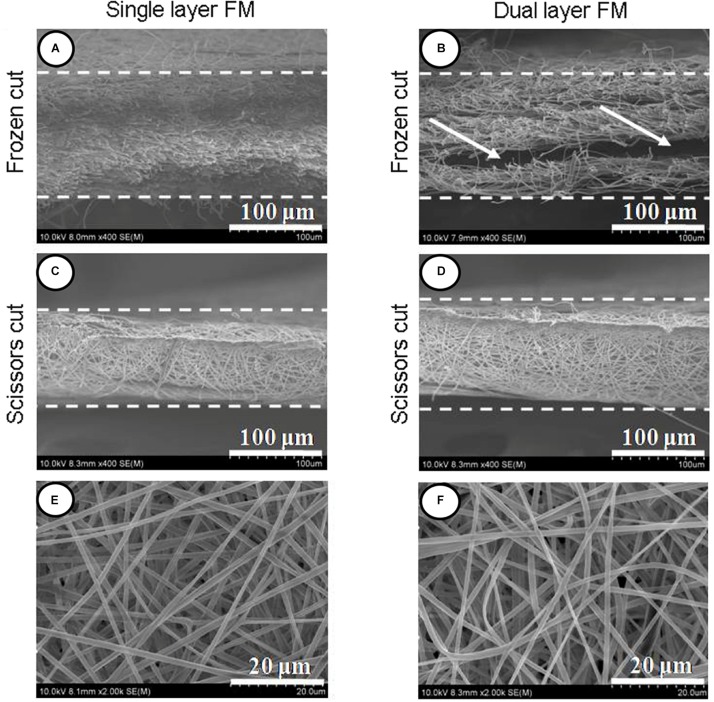
Section view of PLLA electrospun fibrous membranes. **(A)** Single-layer/frozen-cut, **(B)** dual-layer/frozen-cut, **(C)** single-layer/scissor-cut, **(D)** dual-layer/scissor-cut. White arrow shows the space between the two layers in the dual-layer membrane. Fibers of PLLA electrospun membranes **(E,F)**.

**FIGURE 2:**
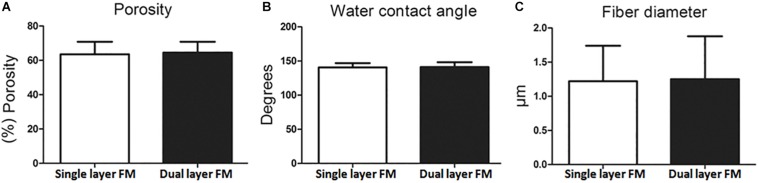
Characteristics of PLLA fibrous membranes. **(A)** Porosity, **(B)** water contact angle, and **(C)** fiber diameter.

Figures 1A–D shows SEM micrographs of single-layer and dual-layer PLLA fibrous membranes before and after scissor cuts. As shown in Figures 1E,F, both types of PLLA fibers were round without beads, porous, and randomly arrayed. The mean diameter of the single-layer PLLA fibers was 1.24 ± 0.54 mm, and that of the dual-layer PLLA fibers was 1.29 ± 0.61 mm. The porosity of the single-layer fibrous membranes was 64%, and that of the double-layer fibrous membranes was 65%. The water contact angles were 142.3° ± 3.8° for the single-layer membrane and 142.8° ± 5.3° for the dual-layer membranes (Figure 2).

### *In vitro* Cell Adhesion and Proliferation

**FIGURE 3:**
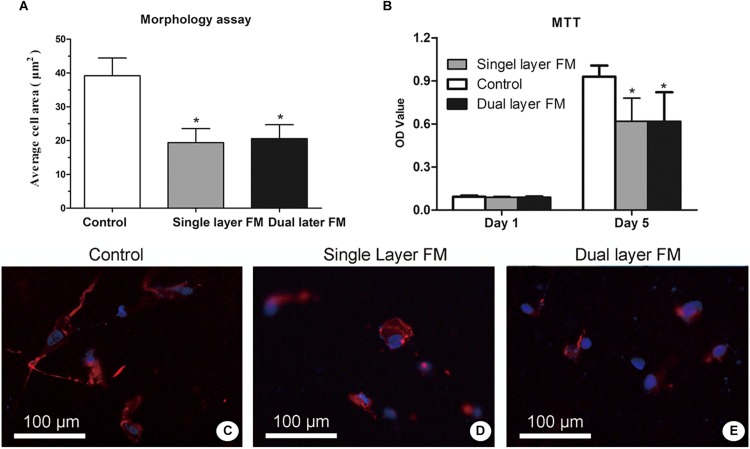
Proliferation **(A)** and toxicity assays **(B)** of fibroblasts on the surface of PLLA fibrous membranes (groups: control, single-layer, and dual-layer, *n* = 3 each). Fluorescence micrographs of chicken embryonic fibroblasts (UMNSAH/DF-1 cells) after 1 day of incubation. The nuclei were stained blue, and the cytoskeleton was stained red on the surface of the fibrous membranes. **(C)** Blank control (tissue culture), **(D)** single-layer PLLA fibrous membranes, and **(E)** dual-layer PLLA fibrous membranes.

Fibroblast proliferation in the single-layer and dual-layer PLLA fiber scaffolds on days 1 and 5 after culture is shown in Figures 3A,B, respectively. Fluorescence micrographs (Figures 3C–E) showed that the fibroblasts adhered less on the control membrane than on either fibrous membrane after a 24-h incubation. This finding indicated that cell growth did not vary between the single-layer and dual-layer PLLA fiber surfaces.

### Animal Implantation Study

**FIGURE 4:**
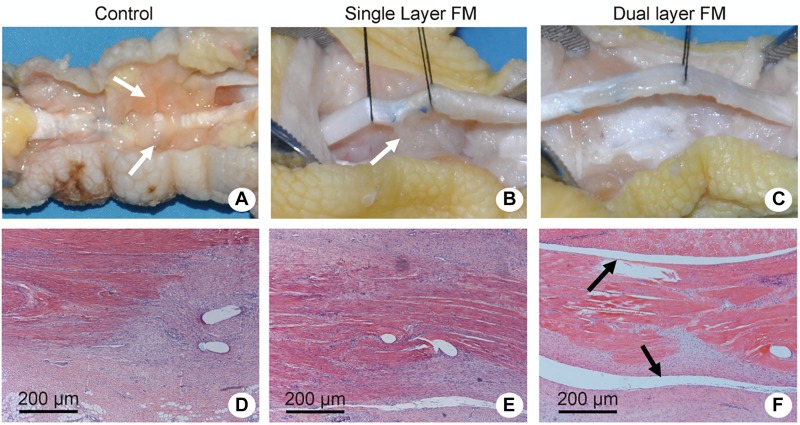
Gross evaluation of tendon repair in chicken models of flexor digitorum profundus tendon injury and Masson’s trichrome/hematoxylin and eosin staining of transverse sections of the repaired tendon sites. Repair sites in **(A,D)** the control group (untreated), **(B,E)** the single-layer PLLA fiber scaffold group, and **(C,F)** the dual-layer PLLA fiber scaffold group. White arrows refer to the adhesive tendons **(A,B)**. Black arrows refer to the peritendinous gliding space **(F)**.

Animal FDP tendons were obtained after 3 weeks of implantation, and the repaired sites were exposed to evaluate peritendinous adhesion and regeneration based on gross morphology (Figure 4). In the control group, highly adhesive tendons were observed in the regenerated areas and were difficult to isolate using dissecting methods (Figure 4A). In the single-layer group, scars connecting the regenerated tendon tissues with the surrounding tissue were also found (Figure 4B), whereas in the dual-layer group, the repair site was smooth (Figure 4C).

**FIGURE 5:**
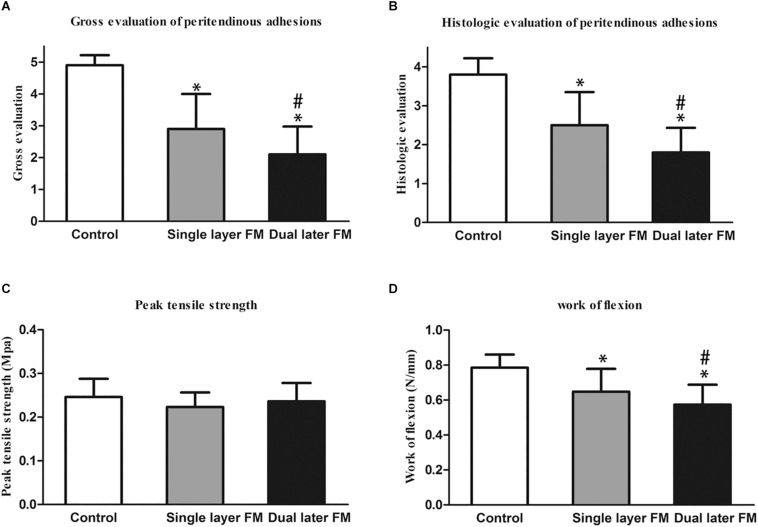
Gross evaluation of the peritendinous adhesions of the sutured flexor digitorum profundus tendon at 3 weeks after operation **(A)** and histological assessment of peritendinous adhesions **(B)**. *P < 0.05 compared with the untreated control group; ^#^*P* < 0.05 compared with the single-layer PLLA fibrous membrane group. Biomechanical tests of the repaired tendons: **(C)** peak tensile strength (force required to pull the flexor digitorum profundus tendon out of the tendon sheath) and **(D)** work of flexion. *P < 0.05 compared with the untreated control group; ^#^*P* < 0.05 compared with the single-layer PLLA fibrous membrane group.

Histologically, the repaired tendons in the control group suggested the presence of a layer of connective tissue in a dense pattern without a peritendinous gliding space (Figure 4D), and some forms of peritendinous adhesion were observed in the single-layer group (Figure 4E). In contrast, no peritendinous adhesion was detected in the dual-layer group (Figure 4F). The findings of gross and histological evaluations of adhesive tendons are presented in Figures 5A,B.

### Biomechanical Analysis

Flexion and peak tensile strength analyses were performed using a rheometer to assess tendon repair and adhesion around tendons. The peak tensile strength showed no significant differences between the three groups (Figure 5C). However, the work of flexion was lowest in the dual-layer fibrous membrane group and highest in the untreated control group (Figure 5D).

## Discussion

Recent studies have focused on drug delivery systems in fibrous membranes for preventing tendon adhesion (Zhao et al., 2015, 2019; Liu W. et al., 2020). Although these systems are functional in animals, their application has been hindered by the complexity of the production process, low efficacy of anti-adhesive agents, poor quality of tendon healing, and uncertainty regarding the effects of drug delivery. Hence, in this study, we developed and investigated the properties of an innovative polymer fibrous membrane to prevent tendon adhesion without drug delivery.

**FIGURE 6:**
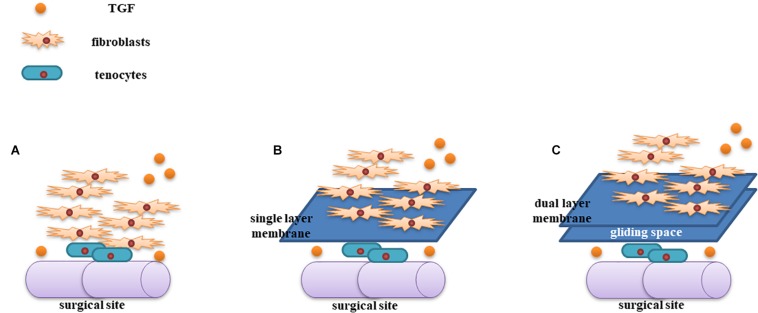
Illustration of the mechanism of the dual-layer PLLA anti-adhesion membrane. TGF-β increases tendon regeneration and fibroproliferation at the surgical site **(A)**. The PLLA membrane could be used to isolate fibroblasts from the repaired tendon **(B)**. The gliding space created by the dual-layer PLLA membrane generated a stabilized tendon **(C)**.

We found several important characteristics of this novel PLLA nanofibrous membrane. First, the microporous structure of PLLA permits exchange of nutrients into the regenerated tendons (Liu et al., 2013) and prevents the detrimental effects of releasing agents from the membrane on the intrinsic healing of tendons. TGF-β is one of the most important cytokines involved in tendon regeneration and tissue adhesion (Liu et al., 2019). Moreover, TGF-β enhances collagen synthesis and fibroblast proliferation via the extracellular signal-regulated kinase 2/SMAD pathway (Liu C. et al., 2020), and increased collagen levels in turn stimulate tendon regeneration. Importantly, TGF-β can localize to repaired tendon sites, resulting in increased collagen synthesis and tendon healing. In our study, we found that the membrane isolated the adhesion caused by TGF-β-induced fibroproliferation (Figure 6). Thus, the strength of the repaired tendon in the PLLA group was comparable to that of the control group.

In this study, the dual-layer design guaranteed the formation of a space between the two layers. During preparation of the material, the first layer was managed using ethylalcohol, causing the layer to be dense and enabling the second layer to be electrospun upon the first layer. Subsequently, the second layer was also managed using ethylalcohol. Our results showed that the space between the fibrous layers could function as an artificial gliding layer (e.g., a tendon sheath) during tendon healing. Additionally, the space persisted when the PLLA fibrous membrane was completely degraded, which was beneficial for our tendon repair model. In gross and histological evaluations, we found that the repaired tendon in the dual-layer PLLA fibrous membrane group showed low levels of adhesion in most samples. The work of flexion in the dual-layer group was the lowest.

Finally, the viscous nature of PLLA, combined with drug-free production, made the dual-payer PLLA fibrous membrane more available for clinical practice. Dual layers converged into a single layer at the margins of the membrane when a shearing force was applied (Biswal and Saha, 2019). Thus, the material could be shaped in any size and placed at the target site. Further studies and clinical translational research are needed to confirm the clinical applications of this material.

## Conclusion

In this study, we developed an innovative polymer fibrous membrane to prevent tendon adhesion without drug delivery. This integrated dual-layer PLLA electrospun membrane could prevent peritendonous adhesion via the gliding space left after degradation of membrane. Because of its viscous nature, the margins of the membrane became a single layer when shearing force was applied. Signaling molecules and nutrients, including TGF-β, could exchange through the PLLA membrane, which could increase collagen levels to stimulate tendon regeneration.

Our results suggested that it was feasible to create the space of a dual-layer membrane with single-layer margins. The adhesion and proliferation of fibroblasts were low on the PLLA fibrous membrane. The gliding space persisted until recovery in chicken extensor flexor tendons *in vivo*. Thus, compared with single-layer membranes, the dual-layer PLLA fiber scaffold reduced adhesion to tissues, and the strength of the repaired tendons in the PLLA group was comparable to that in the control group. Based on these results, we concluded that this innovative PLLA membrane could prevent adhesion and promote gliding during tendon healing.

## Data Availability Statement

All datasets generated for this study are included in the article/supplementary material.

## Ethics Statement

The animal study was reviewed and approved by ethical committee of Xiangya Hospital, Central South University (201908798).

## Author Contributions

WW, YQ, and YL conceived the initial idea, the conceptualization, the study design, and participated in the data extraction and analysis, and revised the manuscript. All authors participated in its design, searched databases, extracted the studies read and approved the final manuscript.

## Conflict of Interest

The authors declare that the research was conducted in the absence of any commercial or financial relationships that could be construed as a potential conflict of interest.
